# Tentorium Cerebelli: the Bridge Between the Central and Peripheral Nervous System, Part 2

**DOI:** 10.7759/cureus.5679

**Published:** 2019-09-17

**Authors:** Bruno Bordoni, Marta Simonelli, Maria Marcella Lagana

**Affiliations:** 1 Cardiology, Foundation Don Carlo Gnocchi, Milan, ITA; 2 Osteopathy, French-Italian School of Osteopathy, Pisa, ITA; 3 Radiology, IRCCS Fondazione Don Carlo Gnocchi Onlus, Milan, ITA

**Keywords:** tentorium cerebelli, fascia, pain, venous circulation, neurological connections, cranio

## Abstract

The tentorium cerebelli is a meningeal portion in relation to the skull, the nervous system, and the cervical tract. In this second part, the article discusses the systematic tentorial relationships, such as the central and cervical neurological connections, the venous circulation and highlights possible clinical alterations that could cause pain. To understand the function of anatomy, we should always remember that every area of the human body is never a segment, but a functional continuum.

## Introduction and background

Cervical neurological connections

The ansa cervicalis characterizes the first cervical roots and connects all anterior cervical nerve exits with the inferior floor of the oral cavity, the trigeminal system, the respiratory control system, and the sympathetic system. The descending branch of the hypoglossal nerve anastomoses with C1, forming the ansa hypoglossi or ansa cervicalis superior [1]. The inferior root of the ansa cervicalis, also known as descendens cervicalis, is formed by ascendant fibers from spinal nerves C2-C3 and occasionally fibers C4, lying anteriorly to the common carotid artery (it passes laterally or medially to the internal jugular vein upon anatomical variations) [1]. Some authors suggest an additional anastomosis with posterior cervical nerves [2]. The ansa cervicalis communicates directly with the nodose ganglion of the vagus nerve, particularly from C1-C2, or with a branch originating directly from the vague with spinal nerves [2]. A variant of this connection to the spinal nerves and the vague is the direct communication with the hypoglossal nerve [2]. The inferior roots of the ansa cervicalis exchange fibers with the phrenic nerve, most likely with its accessory fibers, affecting phrenic innervation areas; a direct intervention of the first cervical roots would support the phrenic action [3]. Sympathetic ganglia are connected to the ansa cervicalis, as well as the spinal trigeminal ganglion [3-4]. According to some authors the dorsal root C1, or occipital nerve, may be absent in 8% of the population, and only a low percentage has an anastomosis with the CN XI, through a branch of C1, known as the nerve of McKenzie [5]. The cervical spinal nerve 1 does not show meningeal or articular branches to the vertebra. Throughout most of the population, the nerve has a small ganglion, close to the intervertebral foramen and medial to the dural tissue, functions of which we have few reliable researches about; according to some authors, it might have a role in the perception of chemoreception variations [5]. This means that the ganglion might perceive inflammatory substances if they accumulated in the myofascial tissue, causing occipital pain [6]. The dorsal branch is strictly connected to the vertebral artery and is posterior to the arch of atlas, aiming to innervate sub-occipital muscles and semispinalis capitis; C1 also carries the scalp sensibility [6-7]. An ascending branch reaches the skin of the upper-posterior portion of the neck and the anterior region of the scalp, travelling with the occipital artery [6]. This branch communicates with C2 and C3. The denticulate ligaments link root C1 with C2 [7]. The nerve C2 or great occipital nerve (GON), originates from the medial branch of the dorsal ramus of C2, travelling posteriorly through the first and second vertebra, communicating with the dorsal root of C3 [8-9]. It ascends towards the scalp at about 4 cm from Inion and between the inferior oblique muscle and the semispinalis capitis. In 45% of the population, it crosses the trapezius or its inferolateral aponeurosis near Inion, where it branches [8-9]. It ascends underneath the skin in a vertical pathway with the occipital artery (the artery is often medial to the nerve), until it reaches the vertex. In this area, it shares its innervation area with the trochlear nerve of the trigeminal. This cervical branch interacts with the nuchal ligament of the trapezius, which relates directly to the dura mater in the atlantooccipital space [10]. Studies demonstrate how a branch of C2 is involved with innervation of trapezius [11]. Within anterior branches of C2 the cutaneous ones, such as the transverse nerve, the lesser occipital nerve and the great auricular nerve, carry the sensation from the anterolateral cervical skin and can cause temporomandibular pain [12]. The transverse cervical nerve, also known as superficial cervical or cutaneous cervical (C2-C3), turns around the posterior border of the edge of sternocleidomastoid (SCM) about halfway, and, passing obliquely forward to the anterior border of the muscle, it perforates the superficial cervical fascia, and divides beneath the platysma to innervate the anterolateral part of the neck [13]. The lesser occipital nerve (C2-C3) ascends along the posterior border of the SCM to spread within the occipital and mastoidal cutaneous area [14]. The great auricular nerve arises from the second and third cervical nerves (C2-C3), winds around the posterior border of the SCM (as the previous two), and runs transversally towards the auricle upon the muscle. The third occipital nerve (C3) is the superficial medial branch of the third cervical dorsal ramus, and it supplies the C2-C3 zygapophysial joint while crossing the joint laterally. Also, it supplies part of the semispinalis capitis muscle by travelling deeply along the muscle course, piercing the splenius and trapezius, before sending anastomotic branches to C2. It becomes cutaneous once exiting the nuchal ligament involving sensitive innervation of a small skin area just underneath the nuchal line [15]. The ramus of C3 receives anastomosis from the superior cervical ganglion of the sympathetic system, and is closely related to the vertebral artery, which sends branches right in proximity of the intervertebral foramen; it also receives anastomosis with C4 nerve root [16].

## Review

Central neurological connections

**Figure 1:**
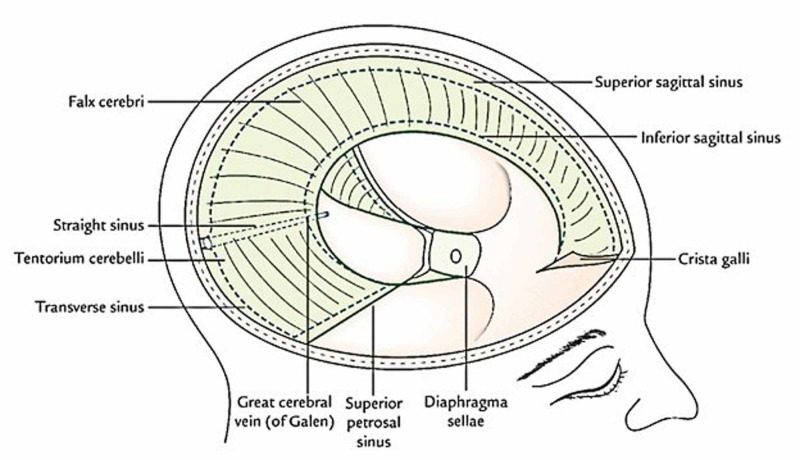
The image shows the dural connections and the venous sinuses.

The fifth cranial nerve or trigeminal nerve is the largest of the cranial nerves. It is responsible for sensation and motor functions in the face, most of the scalp, teeth, oral and nasal cavity, masticatory muscle motor activity and other muscle motor activities [16-17]. It contains proprioceptive fibers from the masticatory and extraocular muscle districts [16]. The trigeminal consists of four mesencephalic nuclei. The mesencephalic nucleus is responsible for transmitting the proprioceptive fibers from the extraocular and masticatory muscles, regulating the bite strength [16]. The afferent fibers to the nucleus carry the pressure and kinesthetic sensations from the teeth, the periodontium, the hard palate, and the temporomandibular joint (TMJ). This nucleus is located in the lower end of the mesencephalon and on the superior pons, laterally to the cerebral aqueduct of sylvius, and along the margins of the periaqueductal gray and anterolateral to the fourth ventricle, medial to the sensory nucleus [16]. The main sensory nucleus carries impulses for the tactile and pressure senses; it is located laterally of the trigeminal root entry zone on the superior pons; its fibers cross the ventral posteromedial nucleus of the thalamus, composing the ventral and dorsal trigeminothalamic tracts [16]. The motor nucleus is medial compared to the sensory nucleus; its fibers exit the brainstem, being then incorporated into the mandibular division, passing below the trigeminal ganglion without creating synapses [16]. The spinal nucleus manages pain and temperature sense modalities and involves the C2-C4 cervical tract until it reaches the anterolateral area of the fourth ventricle. It receives information from the oral and nasal cavity, the facial cutaneous zones, like the cheeks, the forehead, and the jaw [16]. The trigeminal root, also called cisternal segment, is located before entering the Meckel cave and it mainly consists of two-thirds of sensory fibers. It continues forward and below the tentorial border and the superior petrosal sinus, between the meningeal and periosteal layer of the dura mater, finally entering the Meckel cave [16]. This space is a dural fold located on the petrous apex of the temporal bone and closes to the cavernous sinus, containing the trigeminal ganglion (Figure 1). This cave "lays" on the internal carotid [18-19]. The motor and sensory portions entering the Meckel cave are in anastomotic communication, and finally, form the trigeminal ganglion or Gasser’s ganglion [19]. The XII nerve or hypoglossal nerve is considered entirely motor and plays an important role for the tongue [20]. It controls the intrinsic and extrinsic muscles of the tongue (genioglossus, hyoglossus, and styloglossus), and the infrahyoid region through neurological connections with the ansa cervicalis [20]. The XII nerve can be divided based on its anatomical area: intracranial, cisternal, skull base, and extracranial [21]. The first segment is the hypoglossal nucleus, located in the dorsal spinal cord between the midline and the dorsal nucleus of the X cranial nerve; it continues as a thin nucleus with a caudocranial direction to the medulla [21]. The hypoglossal nucleus receives fibers from the glossopharyngeal nerve, the vague and the trigeminal system, in order to mediate a wide variety of stimuli and reflexes coming from the tongue and the pharyngeal mucosa, necessary for swallowing and phonating. Circulation comes from anterior spinal and vertebral arteries [21]. The cisternal segment is formed by the coalescence of roots coming out the spinal cord, generating the XII nerve; it can blend with some fibers of the X nerve, and its fibers pass behind the vertebral artery, before entering the hypoglossal canal [21-22]. The skull base area covered by the XII nerve is the hypoglossal canal, which is a foramen in the occipital bone located below the jugular foramen; it runs obliquely forwards (anterolateral) superiorly to each occipital condyle, while the jugular foramen is lateral from the condyle [21]. The extracranial segment can be further divided into a superior carotid space and anterior space [21]. When the hypoglossal nerve exits the hypoglossal canal, near the atlas, it anastomoses with some branches of the superior cervical ganglion of the sympathetic system, and with a fiber that connects to the first and second ascending cervical branches (C1-C4 or ansa cervicalis or hypoglossal ansa). A descending branch of the hypoglossal nerve (or superior root of the ansa cervicalis) initiates from this loop, and it innervates the infrahyoid muscles, such as the thyrohyoid and geniohyoid muscles [21, 23]. This branch may enter the thorax engaging the parasympathetic and sympathetic trunk and the visceral function of the mediastinum [23]. Within the ansa cervicalis area, the XII nerve communicates with the glossopharyngeal nerve, with the possibility of anastomosis between the two branches of the hypoglossal nerve, prior to the hyoid bone, between the genioglossus muscle above and the geniohyoid muscle below; this anastomosis is known with different names, such as the suprahyoid loop of the hypoglossal nerve or Hyrtl’s suprahyoid ansa [23]. The descending branch of the hypoglossal nerve can anastomose with the phrenic nerve/ansa cervicalis, innervating the sternothyroid muscle, known as Valentin’s anastomosis [23].

Venous circulation

**Figure 2:**
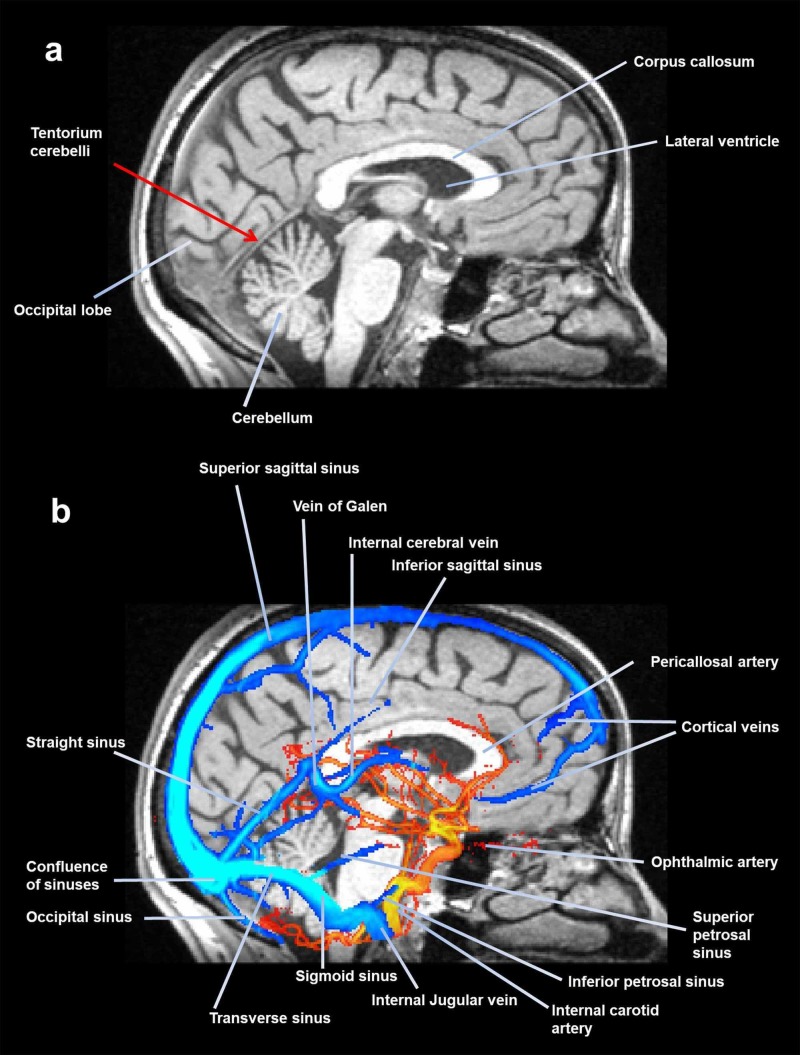
A midsagittal slice of magnetic resonance, where the tentorium is visible. The image shows (above) some portions of the meninges, while the following image (below) shows the venous sinuses and some arteries.

The tentorium cerebelli influences the posterior cranial venous circulation (Figure 2). The superior and inferior sagittal sinuses, and the sinus rectus converge to the torcular herophili, while the occipital sinus can arise from the internal occipital protuberance, which is the insertion of the posterior tentorium cerebelli [24]. A dural structural alteration of the tentorium could cause venous diseases, which may affect the central nervous system and the cervical nervous system [25-28].

## Conclusions

The article discusses the neurological relationships between the tentorium cerebelli and the central and peripheral nervous systems, describing the anatomy of such connections. The supratentorial region is innervated by the trigeminal nerve (V cranial nerve), while the infratentorial region by branches of the cervical plexus, X and XII cranial nerves. The tentorium has fascial relationships with the cervical tract thanks to connections with the sub-occipital muscle, the nuchal and yellow ligaments, and the Hoffman ligaments. The tentorium affects the cerebrospinal fluid and venous circulation.
